# Explainable ensemble machine learning model for prediction of 28-day mortality risk in patients with sepsis-associated acute kidney injury

**DOI:** 10.3389/fmed.2023.1165129

**Published:** 2023-05-18

**Authors:** Jijun Yang, Hongbing Peng, Youhong Luo, Tao Zhu, Li Xie

**Affiliations:** ^1^Department of Critical Care Medicine, Loudi Central Hospital, Loudi, China; ^2^Department of Pulmonary and Critical Care Medicine, Loudi Central Hospital, Loudi, China; ^3^Patient Service Center, Loudi Central Hospital, Loudi, China

**Keywords:** sepsis-associated acute kidney injury, ensemble machine learning, prediction model, XGBoost, MIMIC-IV database

## Abstract

**Background:**

Sepsis-associated acute kidney injury (S-AKI) is a major contributor to mortality in intensive care units (ICU). Early prediction of mortality risk is crucial to enhance prognosis and optimize clinical decisions. This study aims to develop a 28-day mortality risk prediction model for S-AKI utilizing an explainable ensemble machine learning (ML) algorithm.

**Methods:**

This study utilized data from the Medical Information Mart for Intensive Care IV (MIMIC-IV 2.0) database to gather information on patients with S-AKI. Univariate regression, correlation analysis and Boruta were combined for feature selection. To construct the four ML models, hyperparameters were tuned via random search and five-fold cross-validation. To evaluate the performance of all models, ROC, K-S, and LIFT curves were used. The discrimination of ML models and traditional scoring systems was compared using area under the receiver operating characteristic curve (AUC). Additionally, the SHapley Additive exPlanation (SHAP) was utilized to interpret the ML model and identify essential variables. To investigate the relationship between the top nine continuous variables and the risk of 28-day mortality. COX regression-restricted cubic splines were utilized while controlling for age and comorbidities.

**Results:**

The study analyzed data from 9,158 patients with S-AKI, dividing them into a 28-day mortality group of 1,940 and a survival group of 7,578. The results showed that XGBoost was the best performing model of the four ML models with AUC of 0.873. All models outperformed APS-III 0.713 and SAPS-II 0.681. The K-S and LIFT curves indicated XGBoost as the most effective predictor for 28-day mortality risk. The model’s performance was evaluated using ROCpr curves, calibration curves, accuracy, precision, and F1 scores. SHAP force plots were utilized to interpret and visualize the personalized predictive power of the 28-day mortality risk model. Additionally, COX regression restricted cubic splines revealed an interesting non-linear relationship between the top nine variables and 28-day mortality.

**Conclusion:**

The use of ensemble ML models has shown to be more effective than the LR model and conventional scoring systems in predicting 28-day mortality risk in S-AKI patients. By visualizing the XGBoost model with the best predictive performance, clinicians are able to identify high-risk patients early on and improve prognosis.

## Introduction

Sepsis continues to be a major cause of life-threatening conditions in critically ill patients. The excessive pro- or anti-inflammatory response can lead to cellular and organ dysfunction, ultimately resulting in death (1, 2). The most significant sepsis-associated organ disorder is acute kidney injury (AKI), which has a high prevalence (2, 3). AKI is an independent risk factor for high mortality (3, 4), and contributes to 58.6% of the excess attributable mortality (5). Sepsis-associated acute kidney injury (S-AKI) can be caused by microvascular dysfunction, inflammation, and metabolic reorganization. These play a crucial role in the development of S-AKI (3). However, the high heterogeneity in S-AKI is associated with multiple pathogenic mechanisms (3, 4), and there are currently no effective preventive or therapeutic measures available. The treatment for S-AKI is reactive and non-specific, which can result in a high mortality rate due to the difficulty in predicting AKI at the time of patient presentation. As such, salvage therapy is often the primary treatment option (3). However, providing early warning to patients at high mortality risk can help clinicians stratify patient management and improve the prognosis of patients with S-AKI.

Acute kidney injury, is a frequently encountered clinical syndrome that often accompanies critical illness. Its developmental process is complex and multifaceted. It is not sufficient to rely on a single variable to predict the mortality rate associated with AKI. Instead, combining multiple factors would be a more accurate way to forecast the prognosis of AKI (3). In the field of intensive care, conventional scoring systems that integrate clinical symptoms and laboratory data have been extensively utilized to forecast the prognosis of severely ill patients. Notably, the Sequential Organ Failure Assessment (SOFA), Acute Physiology Score III (APS-III), and Simplified Acute Physiology Score II (SAPS-II) scores have demonstrated robust predictive capabilities (6, 7). The prediction of 90-day mortality caused by severe infection-related AKI in China was carried out using COX regression analysis. The study identified several independent predictor variables including age, emergency ICU admission, post-surgical cases, admission diagnosis, AKI etiology, disease severity score, mechanical ventilation, use of boosters and blood outcomes such as albumin, potassium, and pH (8). In a study analyzing 30-day mortality in elderly patients with sepsis, a multivariate logistic regression-based analysis was conducted and resulted in a more accurate prediction with an AUC of 0.831 (9). Additionally, a multivariate prediction model for ICU and in-hospital death in AKI patients undergoing continuous renal replacement therapy found to be more accurate than SOFA, APACHE-II, and SAP-II scores (10). Recent trends suggest that the implementation of big data technologies in healthcare, specifically machine learning, has led to an improvement in the quality of care and optimization of healthcare processes and management strategies (11, 12). Studies have shown that machine learning prediction models have been successful in early warning of AKI occurrence and mortality risk (13, 14), with the XGBoost model achieving a high performance in predicting S-AKI (AUC 0.821) (15). Zhou et al. utilized data from the MIMIC III database to create a machine learning model for predicting AKI within 48 h of sepsis-related ARDS cases. Their model outperformed the discriminatory ability of SOFA (16). This highlights the potential of machine learning algorithms in accurately predicting the development of S-AKI.

Recent studies have shown that machine learning algorithms have achieved better performance in predicting S-AKI prognosis. For instance, the XGBoost model was constructed in a recent study to outperform the SOFA score and SAP-II in predicting mortality at different periods based on dynamic data of S-AKI cases updated every 12 h in the MIMICIV public database (14). However, there is a lack of research comparing multiple ensemble machine learning algorithms for early predict on of the high risk of 28-day mortality in S-AKI. Ensemble ML algorithms differ from traditional prediction models like logistic regression in that they do not involve rigorous screening of variables or adjustment for data imbalance during the construction process. This can lead to overfitting and classification boundary shifting in the resulting models. Previous studies on ML models have not extensively explored the linear or non-linear relationships between significant individual variables of the prediction model and the resulting outcomes.

This project aims to train and test multiple ensemble ML models using S-AKI data from the MIMIC-IV library. The goal is to select the best model that can provide early warning of the 28-day mortality risk in S-AKI cases. The interpretation and visualization of the prediction models are done using SHAP values. Specifically, SHAP force plots are analyzed to identify important mortality-related variables for individual cases. We utilized COX regression-restricted cubic spline plots to analyze the correlation between crucial, independent variables and 28-day mortality. Our ultimate goal is to develop a prediction model that can aid in treatment decisions for patients with S-AKI who are at a high risk of 28-day mortality, ultimately improving their chances of survival.

## Materials and methods

### Participants

The subject case dataset was obtained from the Medical Information Mart Intensive Care IV (MIMIC IV 2.0) database, which provides extensive information on more over 250,000 patients who were admitted to Beth Israel Deaconess Medical Center in Boston, Massachusetts, USA, from 2008 to 2021. The MIMIC IV public database was approved by the Institutional Review Board (IRB) and has undergone a thorough deidentification process. The database is freely available to researchers worldwide after receiving joint approval from the ethics review boards of MIT and Harvard Medical School. Informed consent was waived as the study was retrospective. To request access to the database, one of the investigators (HP) obtained a certificate (certification number 50527660) by passing the Human Research Participant Protection Examination.

### Patients

The study included adult patients aged ≥18 years or older who met the Third International Consensus Definitions for Sepsis and Septic Shock (Sepsis-3) criteria (2), which requires the presence of known or suspected infection along with organ dysfunction wand a Sequential Organ Failure Assessment (SOFA) score of 2 or higher. Additionally, the study also included patients with AKI that was diagnosed and staged according to the 2012 Kidney Disease: Improving Global Outcomes (KDIGO) guidelines. The study excluded patients with renal disease, such as glomerulonephritis, diabetic nephropathy, hypertensive nephropathy, hereditary nephritis, and chronic renal failure caused by various other diseases. Additionally, only the first hospitalization was considered and patients with ICU stays (LOS) of less than 24 h were also excluded.

The study collected a comprehensive set of data on each patient, including their demographics (3 items), vital signs, blood gas analysis, blood cell count, blood biochemistry, hemodialysis phase reduction, and co-morbidities. Additionally, the study recorded information on AKI staging, use of an invasive ventilator, and urine output 24 h after ICU admission, resulting in a total of 60 variables. The study measured disease severity score (SOFA, ASPIII, SAPAII) within 24 h of ICU admission, length of stay, ICU time, 90-day mortality subgroup, in-hospital mortality subgroup, and follow-up time from hospitalization to death. Participants were divided into mortality and groups based on whether death occurred within 28 days.

### Outcomes

The primary outcome after ICU admission was death within 28 days. Secondary outcomes included hospital mortality, length of stayin both the hospital and ICU, and COX regression-restricted cubic spline analysis.

### Statistical methods

In our study, we excluded any variables with missing data greater than 20% of the case data. For the remaining missing values, we used the random forest method to interpolate. We recorded physiological data of patients every hour and used the mean value. For laboratory data, we selected the maximum or minimum value based on the basis that had the greatest impact on outcome in the clinic.

In the baseline data table, continuous variables are presented as median (IQR), and categorical variables as n (%). Appropriate statistical tests such as the Mann–Whitney U test, Student’s t-test, chi-square test, or Fisher’s exact test were used to compare baseline characteristic variables.

In the variable screening process, we first eliminated variables with *P* > 0.05 using univariate logistic regression analysis as they were deemed less likely to be relevant for 28-day mortality. We then removed variables with correlations greater than 0.75 through eliminated by correlation analysis. Finally, we used the “Boruta” package with the random forest algorithm to screen for essential characteristic variables to be included in the final model.

To calculate the lambda value of each variable in the right-skewed distribution, we used the Box-Cox method. We then performed a series of transformations, including square root, inverse, log, and inverse transformations, to obtain the transformed data-set.

**FIGURE 1:**
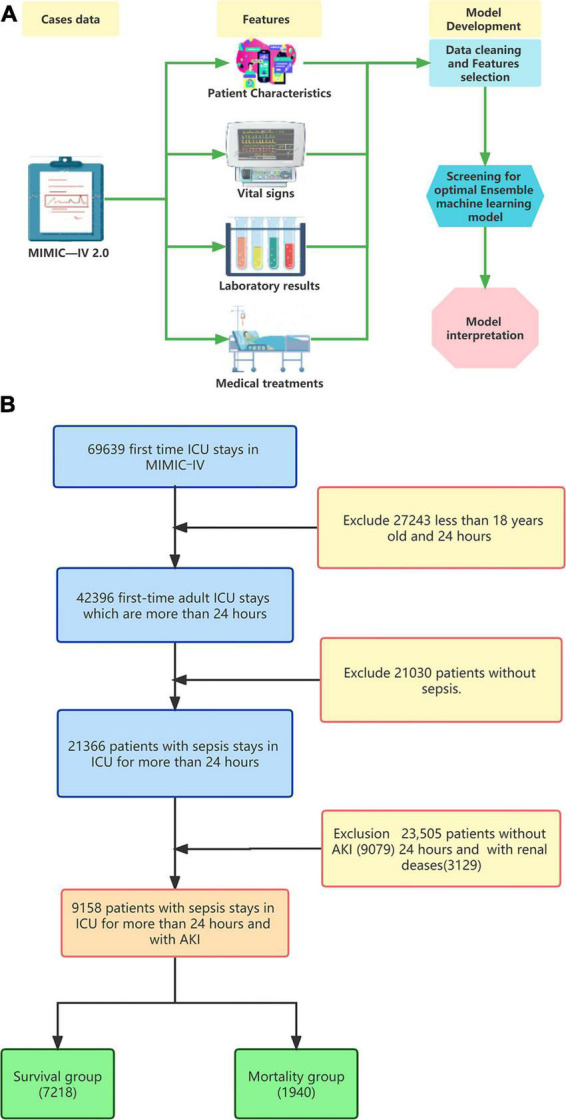
**(A)** Model development process and **(B)** flowchart of the study.

To address data imbalance, we utilized the Synthetic Minority Oversampling Technique (SMOTE) algorithm during the ensemble machine learning model fitting process. The data-set was divided into training and testing sets at a 7:3 ratio. Ensemble learning algorithms, known for their superior performance in machine learning, were employed for the model fitting process. We utilized four models to construct the prediction model: Logistic regression (LR) as the baseline model, and Random Forest (RF), Gradient Boosting Machine (GBM), and Extreme Gradient Boosting Tree (XGBoost) representing the Bagging and Boosting algorithms. The hyperparameters were tuned using the random search method, and the ensemble machine learning model was fitted using the 5-fold cross-validation method. These models were automatically constructed using the ‘creditmodel’ data package. The performance of the four ensemble learning models was evaluated using ROC, K-S, and LIFT curves. AUC values were utilized to compare the differentiation ability of the prediction models with two traditional scoring systems, ASP III and SAPS II, ultimately selecting the best prediction model Additional evaluation of the prediction models’ performance was conducted using ROCpr curves, calibration curves, accuracy, precision, and F1-score. SHapley’s Additional exPlanation (SHAP) is a model-agnostic technique based on cooperative game theory. It is used to explain the predictions filtered through the best ensemble machine learning model. The model construction process was shown in Figure 1A.

The study will also analyze hospital mortality, hospital length of stay, and ICU length of stay as secondary outcomes. In particular, the COX regression analysis will focus on the relationship between important continuous variables and the 28-day risk of death. To analyze the relationship between important continuous data variables and 28-day mortality, we will use COX regression restricted cubic splines with 3 knots. This will be done after adjusting for age and comorbidities, based on the ranking of the most important variables in the prediction model. Both linear and non-linear relationships will be examined.

The analyses were conducted using R version 4.2.1. Our findings are fully reproducible, and the data is available online through the MIMIC-IV(2.0) database.

## Result

### Baseline characteristics

In this study, we extracted data from 69,639 first ICU admissions in MIMIC IV (2.0), and identified 9,158 patients who were diagnosed with sepsis and AKI, had no previous renal disease, and were aged ≥18 years based on nadir criteria. The patients were then divided into two groups: a mortality group (1,940 cases) and a survival group (7218 cases) based on whether they died within 28 days (Figure 1B). The 28-day mortality rate for S-AKI from the MIMIC IV (2.0) dataset was found to be 21.2%. The mortality and survival groups showed significant differences in most baseline variables, as indicated by Tables 1, 2. Patients who died had higher SOFA, APSIII, and SAPS II scores compared to those who survived, as shown in Table 1.

**TABLE 1:** Demographic and clinical characteristics of 28-day survival and mortality group.

	[ALL]	Survival group	Mortality group	*p*-Value
	***N* = 9,158**	***N* = 7,218**	***N* = 1,940**	
**Characteristics (median [IQR] and n (%))**				
Age (years)	67.0 [57.0–78.0]	67.0 [56.0–77.0]	71.0 [59.0–82.0]	<0.001
Gender				<0.001
Female	3,916 (42.8%)	2,992 (41.5%)	924 (47.6%)	
Male	5,242 (57.2%)	4,226 (58.5%)	1,016 (52.4%)	
BMI^a^	29.2 [25.0–34.3]	29.7 [25.4–34.7]	27.6 [23.4–32.7]	<0.001
Urine output (ml)	1,280 [815–1,875]	1,390 [931–1,975]	862 [439–1,415]	<0.001
**Severity score (median [IQR] and mean (SD))^c^**				
APSIII	52.0 [37.0–75.0]	47.0 [34.0–66.0]	79.0 [59.0–101]	0.000
SOFA	3.00 [2.00–5.00]	3.00 [2.00–4.00]	4.00 [2.00–5.00]	<0.001
SAPSII	39.0 [31.0–50.0]	37.0 [29.0–46.0]	50.0 [40.0–61.0]	<0.001
**Vital signs (median [IQR] and mean (SD))**				
Respiratory rate (cpm^d^)	28.0 [24.0–32.0]	27.0 [24.0–31.4]	30.0 [25.0–34.0]	<0.001
Heart rate (cpm^d^)	105 [92.0–120]	103 [91.0–118]	113 [97.0–128]	<0.001
Systolic blood pressure (mmHg)	86.0 [78.0–94.0]	87.0 [79.0–95.0]	82.0 [73.0–92.0]	<0.001
Diastolic blood pressure (mmHg)	44.0 [38.0–50.0]	44.0 [39.0–50.0]	43.0 [36.0–49.0]	<0.001
Mean arterial pressure (mmHg)	57.0 [50.0–63.0]	57.0 [51.0–63.0]	54.0 [47.0–61.0]	<0.001
Temperature (°C)	37.4 [37.0–38.0]	37.4 [37.1–38.0]	37.3 [36.9–38.0]	<0.001
SpO2 (%)	93.0 [90.0–95.0]	93.0 [91.0–95.0]	92.0 [87.0–94.0]	<0.001
ROX index^b^	7.11 [4.89–9.89]	7.18 [5.04–9.83]	6.81 [4.26–10.1]	<0.001
ROX–HR index^b^	6.82 [4.48–9.95]	6.98 [4.71–10.0]	6.12 [3.66–9.55]	<0.001
**Breathing assistance (median [IQR]), n (%)**				
Ventilation				0.001
No vetilation	3,832 (41.8%)	3,087 (42.8%)	745 (38.4%)	
Ventilation	5,326 (58.2%)	4,131 (57.2%)	1,195 (61.6%)	
Dialysis				0.434
No	8,753 (95.6%)	6,892 (95.5%)	1,861 (95.9%)	
Yes	405 (4.42%)	326 (4.52%)	79 (4.07%)	
Dialysis type				0.465
No	8,753 (95.6%)	6,892 (95.5%)	1,861 (95.9%)	
CRRT^e^	248 (2.71%)	196 (2.72%)	52 (2.68%)	
IHD^e^	157 (1.71%)	130 (1.80%)	27 (1.39%)	
In-hospital mortality				0.000
Survival	7,467 (81.5%)	7,103 (98.4%)	364 (18.8%)	
Mortality	1,691 (18.5%)	115 (1.59%)	1,576 (81.2%)	
Recorded time of death (days)	24.0 [7.00–178]	202 [73.0–681]	7.00 [2.00–14.0]	0.000
Hospitalization time (days)	9.17 [5.55–16.5]	9.84 [6.05–17.8]	6.88 [3.07–12.6]	<0.001
ICU time (days)	3.43 [1.92–7.11]	3.24 [1.86–6.92]	4.16 [2.20–7.94]	<0.001
AKI stage^f^				<0.001
1	2873 (31.4%)	2382 (33.0%)	491 (25.3%)	
2	4765 (52.0%)	3789 (52.5%)	976 (50.3%)	
3	1520 (16.6%)	1047 (14.5%)	473 (24.4%)	
**Comorbidities**				
Myocardial infarct				0.054
No	7,636 (83.4%)	6,047 (83.8%)	1,589 (81.9%)	
Yes	1,522 (16.6%)	1,171 (16.2%)	351 (18.1%)	
Congestive heart failure				0.011
No	6,792 (74.2%)	5,397 (74.8%)	1,395 (71.9%)	
Yes	2,366 (25.8%)	1,821 (25.2%)	545 (28.1%)	
Peripheral vascular disease				0.128
No	8,103 (88.5%)	6,367 (88.2%)	1,736 (89.5%)	
Yes	1,055 (11.5%)	851 (11.8%)	204 (10.5%)	
Cerebrovascular disease				<0.001
No	7,958 (86.9%)	6,370 (88.3%)	1,588 (81.9%)	
Yes	1,200 (13.1%)	848 (11.7%)	352 (18.1%)	
Dementia				<0.001
No	8,808 (96.2%)	6,994 (96.9%)	1,814 (93.5%)	
Yes	350 (3.82%)	224 (3.10%)	126 (6.49%)	
Chronic pulmonary disease				0.372
No	6,806 (74.3%)	5,380 (74.5%)	1,426 (73.5%)	
Yes	2,352 (25.7%)	1,838 (25.5%)	514 (26.5%)	
Rheumatic disease				1.000
No	8,841 (96.5%)	6,968 (96.5%)	1,873 (96.5%)	
Yes	317 (3.46%)	250 (3.46%)	67 (3.45%)	
Peptic ulcer disease				0.075
No	8,890 (97.1%)	7,019 (97.2%)	1,871 (96.4%)	
Yes	268 (2.93%)	199 (2.76%)	69 (3.56%)	
Mild liver disease				<0.001
No	7,610 (83.1%)	6,201 (85.9%)	1,409 (72.6%)	
Yes	1,548 (16.9%)	1,017 (14.1%)	531 (27.4%)	
Diabetes mellitus without complications				0.015
No	6,934 (75.7%)	5,424 (75.1%)	1,510 (77.8%)	
Yes	2,224 (24.3%)	1,794 (24.9%)	430 (22.2%)	
Diabetes mellitus with complications				0.023
No	8,737 (95.4%)	6,867 (95.1%)	1,870 (96.4%)	
Yes	421 (4.60%)	351 (4.86%)	70 (3.61%)	
Paraplegia				<0.001
No	8,755 (95.6%)	6,935 (96.1%)	1,820 (93.8%)	
Yes	403 (4.40%)	283 (3.92%)	120 (6.19%)	
Malignant cancer				<0.001
No	7,917 (86.4%)	6,385 (88.5%)	1,532 (79.0%)	
Yes	1,241 (13.6%)	833 (11.5%)	408 (21.0%)	
Severe liver disease				<0.001
No	8,314 (90.8%)	6,684 (92.6%)	1,630 (84.0%)	
Yes	844 (9.22%)	534 (7.40%)	310 (16.0%)	
Metastatic solid tumor				<0.001
No	8,586 (93.8%)	6,933 (96.1%)	1,653 (85.2%)	
Yes	572 (6.25%)	285 (3.95%)	287 (14.8%)	
AIDS				0.487
No	9,117 (99.6%)	7,188 (99.6%)	1,929 (99.4%)	
Yes	41 (0.45%)	30 (0.42%)	11 (0.57%)	

Continuous variable data are presented as median (SD or interquartile ranges, IQR). Classified variable data are presented as n (%). Unless otherwise stated, the Mann–Whitney U test is used for the continuous variable, the χ^2^ test, or the Fisher’s exact test for the categorical variable.

^a^BMI, body mass index.

^b^ROX, ratio of SpO2/FIO2 to respiratory rate; ROX-HR, the ratio of ROX index over HR (beats/min), multiplied by a factor of 100.

^c^APSIII, Acute Physiology Score III; SAPSII, Simplified Acute Physiology Score II; SOFA, Sequential Organ Failure Assessment.

^d^cpm, counts per minute.

^e^CRRT: continuous renal replacement therapy; IHD: Intermittent Hemodialysis.

^f^AKI, acute kidney injury.

**TABLE 2:** Laboratory results of all patients within 24 h after admission to ICU.

	[ALL]	Survival group	Mortality group	*p*-Value
	***N* = 9158**	***N* = 7218**	***N* = 1940**	
**Arterial blood gas analysis (median [IQR] and mean (SD))**				
pH	7.31 [7.24–7.36]	7.31 [7.26–7.36]	7.28 [7.17–7.36]	<0.001
PaO_2_ (mmHg)	75.0 [46.0–103]	80.0 [52.0–108]	55.0 [39.0–84.0]	<0.001
PaCO_2_ (mmHg)	47.0 [41.0–54.0]	47.0 [42.0–54.0]	47.0 [40.0–57.0]	0.384
PaO_2_/FiO_2_ ratio	168 [100–248]	175 [108–254]	134 [78.0–226]	<0.001
Base excess (mmol/L)	−3.00 [−7.00 to 0.00]	−3.00 [−6.00 to 0.00]	−5.00 [−10.00 to 0.00]	<0.001
Lactate (mmol/L)	2.40 [1.70–3.80]	2.40 [1.60–3.50]	3.10 [1.80–5.90]	<0.001
Anion gap (mmol/L)	16.0 [13.0–19.0]	15.0 [13.0–18.0]	18.0 [15.0–23.0]	<0.001
Bicarbonate (mmol/L)	24.0 [22.0–26.0]	24.0 [22.0–26.0]	23.0 [20.0–26.0]	<0.001
**Complete blood cell count (median [IQR])**				
White cell count (× 109/L)	14.8 [10.8–19.8]	14.6 [10.8–19.3]	15.8 [10.9–21.8]	<0.001
Neutrophil count (× 10^9^/L)	8.87 [5.12–13.6]	8.57 [5.01–13.0]	10.3 [5.75–15.9]	<0.001
Eosinophils count (× 10^9^/L)	0.06 [0.01–0.15]	0.07 [0.01–0.16]	0.03 [0.00–0.11]	<0.001
Lymphocyte count (× 10^9^/L)	1.02 [0.58–1.65]	1.07 [0.62–1.71]	0.87 [0.47–1.42]	<0.001
Monocytes count (× 10^9^/L)	0.53 [0.31–0.85]	0.52 [0.31–0.83]	0.59 [0.34–0.97]	<0.001
Platelets count (× 10^9^/L)	155 [105–218]	155 [109–216]	154 [86.0–229]	0.018
NLR ratio^a^	7.88 [3.99–15.9]	7.33 [3.80–14.7]	10.5 [5.03–21.1]	<0.001
PLR ratio^a^	145 [77.8–281]	140 [76.5–274]	167 [81.2–318]	<0.001
LMR ratio^a^	1.90 [0.96–3.53]	2.00 [1.00–3.75]	1.44 [0.72–2.75]	<0.001
Hemoglobin (g/L)	9.80 [8.30–11.3]	9.80 [8.40–11.3]	9.60 [7.97–11.4]	<0.001
**Blood chemistry results (median [IQR] and mean (SD))**				
Blood glucose (mg/dl)	101 [86.0–124]	100 [86.0–121]	107 [85.0–134]	<0.001
Albumin (mg/dl)	3.20 [2.60–3.80]	3.30 [2.70–3.90]	3.00 [2.40–3.60]	<0.001
Blood urea nitrogen (mmol/L)	21.0 [16.0–32.0]	20.0 [15.0–29.0]	30.0 [20.0–46.0]	<0.001
Creatinine (mg/dl)	1.10 [0.80–1.60]	1.00 [0.80–1.40]	1.40 [1.00–2.20]	<0.001
**Blood chemistry results (median [IQR])**				
Calcium (mmol/L)	7.90 [7.40–8.40]	8.00 [7.40–8.40]	7.80 [7.10–8.40]	<0.001
Chloride (mmol/L)	103 [99.0–106]	104 [100–107]	101 [97.0–105]	<0.001
Sodium (mmol/L)	137 [134–140]	137 [135–139]	137 [133–140]	<0.001
Potassium (mmol/L)	4.50 [4.10–5.00]	4.50 [4.10–4.90]	4.60 [4.10–5.30]	<0.001

Continuous variable data are presented as median (SD or interquartile ranges, IQR). Classified variable data are presented as n (%). Unless otherwise stated, the Mann–Whitney U test is used for the continuous variable, the χ^2^ test, or the Fisher’s exact test for the categorical variable.

^a^NLR, neutrophil to lymphocyte ratio; PLR, platelet to lymphocyte ratio; LMR, lymphocyte to monocyte ratio.

### Data cleaning and features selection

In our study, we excluded variables with missing values greater than 20%. For the remaining variables, we used the random forest method to perform multiple interpolations on the missing values (Supplementary Figure 1). The interpolation density plot demonstrated that the five interpolated datasets closely matched the distribution of the original data set (Supplementary Figure 2). The complete data set, consisting of 60 independent variables, was obtained after selecting the best-interpolated data set.

**FIGURE 2:**
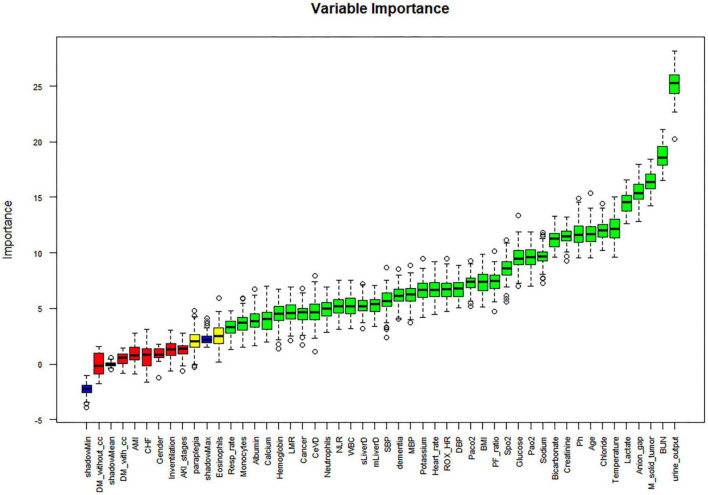
Boruta-based feature selection results. DM-without-cc, diabetes mellitus without complications; DM-with- cc, diabetes mellitus with complications; AMI, acute myocardial infarction; CHF, congestive heart failure; LMR, lymphocyte to monocyte ratio; CeVD, cerebrovascular disease; NLR, neutrophil to lymphocyte ratio; SBP, systolic blood pressure; DBP, diastolic blood pressure; MBI, body mass index; ROX_HR, the ratio of ROX index over HR (beats/min), multiplied by a factor of 100; PF_ratio, PaO2/FiO2 ratio; M_solid_tumor, Metastatic solid tumor; BUN, blood urea nitrogen.

Univariate regression analysis was performed for all variables, and those with a *p*-value greater than 0.05 were removed. The following variables were excluded: ROX, Platelets, Basophils, Lymphocytes, PLR, Peripheral vascular disease, Chronic pulmonary disease, Rheumatic disease, Peptic ulcer disease, AIDS, Dialysis, and Dialysis type. The study conducted a univariate regression analysis on all variables and presented the results using forest plots (Supplementary Figure 3). In the correlation study of continuous variables, those with a correlation greater than 0.75 were eliminated, leaving only the variable with the most significant impact on 28-day death. As a result, Base excess was eliminated (Supplementary Figure 4). The Boruta algorithm, which is based on random forest, was used to sort the importance of variables for further variable screening. This resulted in the identification of 38 variables that were deemed appropriate for model fitting (as shown in Figure 2). Prior to fitting the machine learning model, data distribution analysis was performed on all continuous variables, and box plots were obtained (as demonstrated in Supplementary Figure 5A). To address right-skewed distribution, we utilized the Box-Cox method to calculate the lambda value for each variable. For variables with a lambda value close to -0.5, such as BMI, Pao2, Lactate, Creatinine, BUN, and Anion gap, we performed a square root inverse conversion. We performed log conversion for the lambda values of Glucose, LMR, Pao2/Fio2 ratio, Urine output, WBC, Neutrophils, NLR, ROX-HR, and Monocytes, which were close to 0. For Bicarbonate, which had a lambda value close to 0.5, we performed square root conversion. The lambda value of Respiration rate, Paco2, and Potassium were close to -1, so we performed log conversion for these variables as well. The effect after the transfer was shown by a box plot (as demonstrated in Supplementary Figure 5B). The transformed data were integrated with other untransformed data to create a new dataset for building and internally validating the model.

### Development of 28-day mortality risk prediction model

**FIGURE 3:**
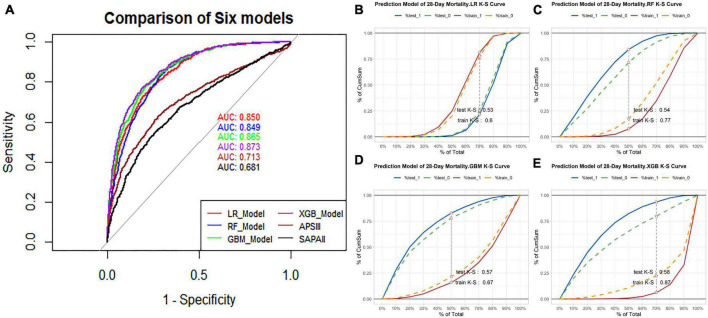
**(A)** Using receiver operating characteristic (ROC) curve and area under the receiver operating characteristic curve (AUC) to compare the discriminant ability of four models and traditional scoring. **(B)** K-S curve of 28-day mortality risk prediction model based on Logistic regression, test K-S 0.53 and train K-S 0.6. **(C)** K-S curve of 28-day mortality risk prediction model based on the Random Forest, test K-S 0.54 and train K-S 0.77. **(D)** K-S curve of 28-day mortality risk prediction model based on the GBM, test K-S 0.57 and train K-S 0.67. **(E)** K-S curve of 28-day mortality risk prediction model based on the XGBoost, test K-S 0.58 and train K-S 0.87.

**FIGURE 4:**
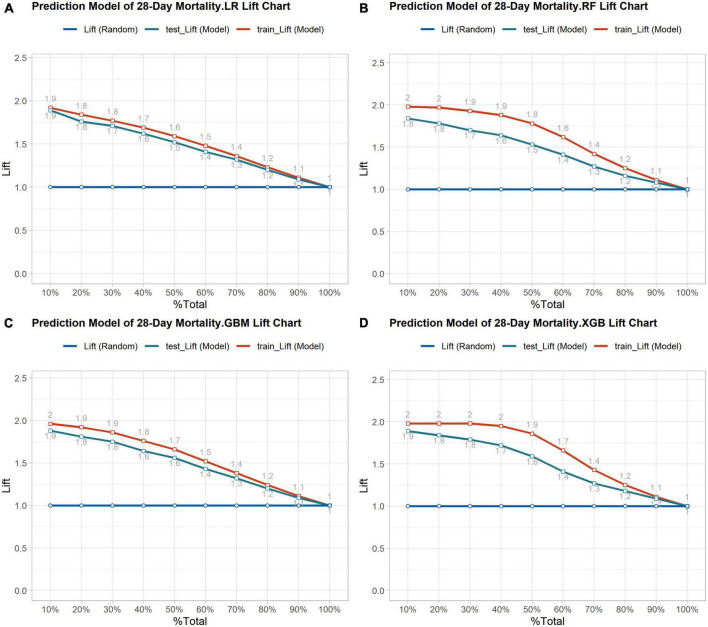
**(A)** Lift curve of 28-day mortality risk prediction model based on Logistic regression. **(B)** Lift curve of 28- day mortality risk prediction model based on the Random Forest. **(C)** Lift curve of 28-day mortality risk prediction model based on the GBM. **(D)** Lift curve of 28-day mortality risk prediction model based on the XGBoost.

Out of the total number of patients, 1,940 individuals passed away within 28 days, resulting in a mortality rate of 21.2% in the dataset. The balanced dataset was created using the SMOTE algorithm and then divided into a training and testing set with a ratio of 7:3. The AUC values for the four prediction models in the testing set were as follows: XGBoost model had an AUC value of 0.873 (with a range of 0.860-0.886), GBM model had an AUC value of 0.865 (with a range of 0.851-0.878), RF model had an AUC value of 0.849 (with a range of 0.834-0.863), and LR model had an AUC value of 0.850 (with a range of 0.836-0.864). The study found that all four machine learning models performed similarly and were more accurate than the traditional scoring systems ASPIII (0.713 95% CI 0.694-0.733) and SAPS II (0.681 95% CI 0.661-0.701). The ROC curve analysis demonstrated that the ensemble machine learning algorithm was significantly better than outperforms the traditional scoring system in predicting the 28-day mortality risk (as shown in Figure 3A). The K-S curves depicted in Figures 3B–E indicate that XGBoost exhibits a slightly superior differentiation ability compared to the other prediction models. Additionally, the LIFT curve (Figure 4) demonstrates that XGBoost outperforms the other models in the 40-50% position of the testing set. This could be attributed to XGBoost’s algorithm, which has demonstrated exceptional learning performance in tabular data, and its robustness to noise, which is attributed to its regularization technique. The ensemble machine learning algorithm, XGBoost, was selected to build the 28-day mortality risk prediction model for S-AKI.

### XGBoost model optimization and visualization

**FIGURE 5:**
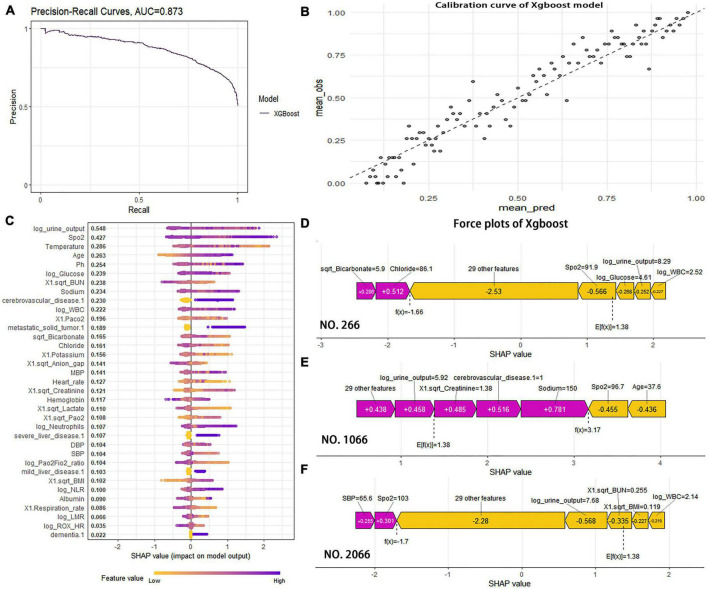
**(A)** The precision-recall (PR) curve was used further to evaluate the classification ability of the XGBoost model; AUCPR (0.873) indicated the model performed well in predicting case classification. **(B)** The calibration curve showed high coherence between the predicted and actual probability of XGBoost model. **(C)** The features are ranked according to the sum of the SHAP values for all patients, and the SHAP values are used to show the distribution of the effect of each feature on the XGBoost model outputs. **(D)** The sharp value force plot of case 266 was used to individually predict the characteristic variables. **(E)** The sharp value force plot of case 1066 was used to individually predict the characteristic variables. **(F)** The sharp value force plot of case 2066 was used to individually predict the characteristic variables.

The XGBoost model was optimized and evaluated using the “xgboost” package. The area under the precision-recall curve (AUCpr) was found to be 0.873, which was similar to the area under the ROC curve (Figure 5A). This suggests that the model has comparable predictive ability for both death and survival. The model’s accuracy, precision, recall, and F1-score were 0.773, 0.724, 0.896, and 0.801, respectively. The results indicate that the XGBoost model performed well in predicting mortality and survival groups. Additionally, the Recall metric outperformed Accuracy, which minimizes the possibility of under diagnosing mortality cases. The calibration curve analysis demonstrated that the model was accurately calibrated for predicting 28-day mortality risk, with no significant overestimation or underestimation (Figure 5B).

To determine the contribution of each variable to the XGBoost model, SHAP values were utilized. The importance of each feature was calculated using the Shapley value, which compared the model’s prediction with and without the feature using the “shapviz” package (Figure 5C). The logarithm of urine output during the first 24 h of ICU admission was found to be the most important variable in predicting the 28-day mortality risk in patients with S-AKI. Among the important variables, pulse oxygen, temperature, age, and pH et al. are included. Cerebrovascular disease is one of the most significant comorbidities that affect the risk of death within 28 days. In Figures 5D–F, SHAP explanatory force plots were used to analyze three cases in the test group (#266, #1066, and #2066), Each variable’s Shapley value is represented by an arrow that indicates an increase (red positive values) or decrease (yellow negative values) in the prediction. The force plots also show the main variables and their corresponding values. The variables that have a significant influence on the prediction vary from case to case.

### Secondary outcomes

Our analysis of essential patient information revealed that the in-hospital mortality rate of S-AKI was 18.2%. Of these patients, 81.2% died within 28 days, with the primary time of death occurring within this timeframe. Additionally, 364 cases (18.8%) resulted in death within 28 days after discharge from the hospital. The death group had a shorter hospitalization duration compared to the survival group, by three days (6.88 [3.07–12.6] vs. 9.84 [6.05–17.8]). However, the death group had a slightly longer duration of ICU stay compared to (4.16 [2.20–7.94] vs. 3.24 [1.86–6.92]). It was observed that the severity of S-AKI condition was directly proportional to the length of ICU stay and increased the risk of early death.

**FIGURE 6:**
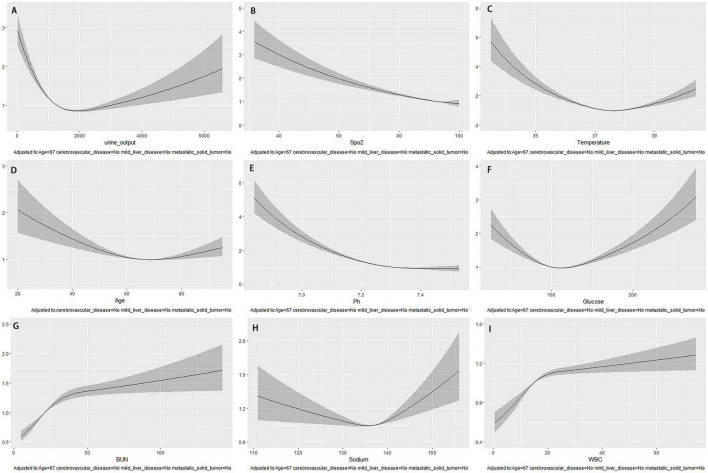
After adjusting for age and underlying disease, the COX regression-restricted cubic spline examined the nonlinear relationship between nine continuous variables and 28-day mortality risk. **(A)** Urine output within the first 24 h; **(B)** Spo2; **(C)** temperature; **(D)** age; **(E)** Ph; **(F)** glucose; **(G)** BUN; **(H)** sodium; **(I)** WBC.

The study found that the independent prediction performance of the top nine continuous variables in the XGBoost model for 28-day death risk was unclear. To detect non-linear or linear relationships between these variables and 28-day mortality, restricted cubic splines of COX regression were used. The model was adjusted for age (67 years) and comorbidities such as cerebrovascular disease, mild liver injury, and metastatic solid tumors. The results are presented in Figure 6. The study found that SpO2 and pH had a nearly linear relationship with a higher risk of death associated lower values. Additionally, variables such as 24-h urine volume (approximately 1500 ml), temperature (approximately 37.3°C), age (approximately 67 years), glucose (approximately 100 mg/dl), and sodium (approximately 136 mmol/L) showed a U-shaped change, with the risk of death being higher at the highest or lowest values relative to the bottom of the curve. The initial levels of BUN (around 37 mg/dl) and WBC (around 20 × 10^9^/L) showed a steep increase, but later on, they remained relatively stable. Moreover, there was no significant rise in the mortality risk with the increase in these values.

## Discussion

Acute kidney injury is a significant contributor to high mortality rates in sepsis patients. Early recognition and management are crucial in preventing the need for salvage treatment and reducing mortality. However, traditional scoring systems do not adequately meet clinical needs. This study proposes using machine learning to predict the 28-day risk of death from S-AKI in ICU inpatients, providing personalized predictions to guide clinical stratification and grading management. The risk of death in these patients has been a challenging aspect to predict in the past.

Clinical symptoms and laboratory tests are frequently employed in traditional scoring systems to predict critical patient outcomes. Two representative methods are the ASP III and SAPS II scores, both of which exhibit strong performance in predicting in-hospital patient mortality (17–19). Previous research has indicated that traditional scores were slightly less reliable in predicting hospitalization due to Acute Kidney Injury (AKI) or mortality within 60 days (6, 7). And it has not been used to predict death within 28 days. In recent years, there has been a growing interest in utilizing machine learning (ML) algorithms for diagnostic and prognostic disease studies. These ML models have shown to surpass traditional scoring methods in terms of predictive accuracy (15, 16). In our study, we also observed that machine learning models outperformed conventional scoring systems in all 28-day mortality prediction for S-AKI patients. The XGBoost algorithm-based 28-day mortality risk prediction model for S-AKI achieved better prediction performance with an AUPR value of 0.873 and good calibration performance. Our XGBoost model demonstrated a slightly better predictive performance compared to another study that utilized the same database (MIMIC-IV), study endpoint and ML algorithm. The area under the curve (AUC) was 0.850, while the other study achieved an AUC of 0.818 (14). Our model’s superior performance of our model may be attributed to the inclusion of co-morbidities in our predictor variables. It is known that cases with co-morbidities have a higher mortality rate in patients with sepsis. Compared with the traditional scoring system, The use of machine learning prediction models can potentially enhance clinicians’ decision-making and improve disease prognosis.

The most critical step in training machine learning models is data engineering, particularly data preprocessing. This process plays a vital role in preventing the risk of overfitting and classification boundary shifts, ultimately leading to improved predictive performance of the models. Despite the importance of data preprocessing, it is often overlooked, and most machine learning models still require thorough investigation in this area (14–16). The S-AKI model we created to predict 28-day mortality risk underwent thorough data processing. We utilized a combination of univariate regression, correlation analysis, and variable screening with Boruta of the random forest algorithm. The Boruta algorithm is a powerful and robust variable screening method that is sensitive to detecting causal variables while minimizing the number of false positives, making it suitable for both high-dimensional and low-dimensional datasets (20). When working with unbalanced categorical datasets, machine learning algorithms may not be reliable and their predictions may be biased, leading to misleading accuracy. To address this issue, we apply the SMOTE algorithm to discard the practice of randomly oversampling replicate samples, which can prevent the problem of random oversampling prone to overfitting. Studies have shown that this approach can improve classifier performance (21, 22). The synthetic data algorithm addresses the issue of data imbalance by avoiding information loss in both undersampling and oversampling methods.

Structured data dominates medical databases, and XGBoost has emerged as a top-performing integrated machine learning algorithm for prediction and classification based on this data (15, 16, 23). Hou, et al. (24) utilized MIMIC III (V1.4) sepsis patient data to develop an algorithm based on XGBoost for predicting 30-day mortality in septic patients. Their algorithm outperformed the logistic regression model and SAPS-II score prediction model with an AUC of 0.857 compared to 0.819 and 0.797, respectively. Additionally, the XGBoost algorithm demonstrated superior accuracy for sepsis diagnosis compared to the SOFA score with an AUC of 0.89 versus 0.596 (25). Liu, J and colleagues (26) utilized eICU data to develop a mortality prediction model for ICU AKI patients. Their study found that the XGBoost model outperformed LR, SVM, and RF machine learning algorithms. Previous research has demonstrated the efficacy of XGBoost as an ensemble machine learning algorithm in disease diagnosis and prognosis studies, particularly structured data. In this study, the performance of RF based on Bagging ensemble machine learning algorithm and XGBoost and GBM based on Boosting method were compared to traditional logistic regression in predicting 28-day mortality in S-AKI. The results indicated that the ensemble learning algorithms outperformed logistic regression. Among the ensemble algorithms, XGBoost demonstrated the best performance, as evidenced by the ROC, K-S, and LIFT curves.

The prediction model for 28-day mortality risk characteristics was ranked using SHAP values, with the logarithm of the 1st 24-h urine volume being identified as the most important variable. According to a study conducted on 183 intensive care units in Australia and New Zealand (27), a urine output threshold of less than 0.5 ml/kg/h within 24 h of ICU admission was found to be predictive of mortality in intensive care unit patients. Furthermore, the study trained an XGBoost machine learning model to predict in-hospital mortality, and discovered that low urine output was strongly associated with mortality in patients with sepsis. In patients with S-AKI receiving continuous renal replacement therapy (CRRT), urine output within the first 24 h of CRRT initiation was found to be a significant predictor of death (HR 2.6 95% CI 1.6–4.3 *p* < 0.001)among the various clinical variables related to mortality (28). Our study revealed that the logarithmic value of urine volume within the first 24 h is closely linked with the highest weight in the 28-day mortality risk model. Additionally, utilizing COX regression-restricted cubic splines and adjusting for age and underlying disease, we discovered a non-linear relationship between 24-h urine volume and 28-day mortality risk. The inflection point was observed at a 24-h urine volume of approximately 1,800 ml. Below this threshold, the risk of death decreased as urine volume increased, while above it, the risk of death increased with increasing urine volume.

Previous research has established that SpO2 is a risk factor for sepsis-related death (29). Similarly, our study discovered that SpO2 was linked to a higher likelihood of 28-day mortality in S-AKI cases. Using COX regression-restricted cubic splines study, we observed a near-linear negative correlation between SpO2, pH, and the risk of 28-day mortality. The relationship between temperature, age, glucose, BUN, sodium, and WBC and 28-day mortality risk was found to be non-linear. Specifically, body temperature, age, blood glucose, and sodium ions showed U-shaped changes, while BUN and WBC exhibited a post-phase plateau.

The variables that determine death risk differ between cases due to their non-linear relationship. In our study, SHAP force plots provide a direct graphical illustration for ensemble learning visualization interpretation. The color yellow represents a negative association with 28-day mortality risk, while red represents a positive association. The ability of machine learning predictions to show individualization is further illustrated by the fact that the variables that play a significant role in three different cases are not perfectly correlated. In some cases, the same variable may have opposite effects, such as the logarithmic value of 24-h urine volume, which is negatively correlated in #266 and #2066 and positively correlated in #1066. This may be due to a U-shaped relationship between urine volume and the risk of 28-day death.

### Limitations

While this study provides valuable insights, it is important to acknowledge its limitations. It is a single-center retrospective data modeling study that relies solely on the MIMIC-IV (2.0) database and lacks external validation. Future studies will incorporate a multicenter dataset and prospective study data to optimize and externally validate the model. Second, it is important to consider that there may be other factors that can affect the 28-day mortality risk in patients with S-AKI that were not measured or extracted, such as imaging data and treatment strategy. To improve the accuracy of predictive models, it may be beneficial to incorporate different types of data and use multimodal algorithms. Third, the data engineering process involves several steps, including data interpolation, feature selection, variable transformation, and data imbalance processing. However, these steps can sometimes lead to model overfitting and misrepresentation of important features. In our next study, we will focus on ensuring the completeness of the data set. Additionally, different types of variables are sequentially incorporated into the construction of the model to observe the effects of different variables on the prediction performance of the model. Finally, we utilize two integration algorithms, bagging and Boosting, and may introduce stacking integration algorithms in the future.

## Conclusion

In this study, we have showcased the effectiveness of ensemble machine learning algorithms in predicting the risk of mortality within 28 days of patients with S-AKI. The SHAP approach has been used to enhance the interpretability of these models, thereby enabling clinicians to gain a better understanding of the underlying reasons behind the results. This knowledge will aid clinicians in making informed clinical decisions with regard to the stratification and management of S-AKI patients.

## Data availability statement

The original contributions presented in this study are included in the article/Supplementary material, further inquiries can be directed to the corresponding author.

## Ethics statement

The studies involving human participants were reviewed and approved by the Institutional Review Boards of the Beth Israel Deaconess Medical Center and Massachusetts Institute of Technology. Written informed consent for participation was not required for this study in accordance with the national legislation and the institutional requirements.

## Author contributions

JY, HP, and LX conceived the study and designed the trial. All authors involved in data collection, data management, data quality control, and data statistical analysis, participated in the revision of the manuscript, and reviewed and approved the final manuscript.
