# Supplementary material for: Explainable ensemble machine learning model for prediction of 28-day mortality risk in patients with sepsis-associated acute kidney injury
Source: Front Med (Lausanne). 2023 May 18;10:1165129. doi: 10.3389/fmed.2023.1165129 (PMC10232880; doi:10.3389/fmed.2023.1165129)
Supplement: Supplementary file 1 [file Data_Sheet_1.doc]

Supplementary Information

1. Supplementary Figure S1 Number of Missing Values

2. Supplementary Figure S2 Interpolation data distribution map

3. Supplementary Figure S3 The forest plots of univariate regression analysis

4. Supplementary Figure S4 Correlation heat map between continuous variables

5. Supplementary Figure S5 Variable conversion box diagram


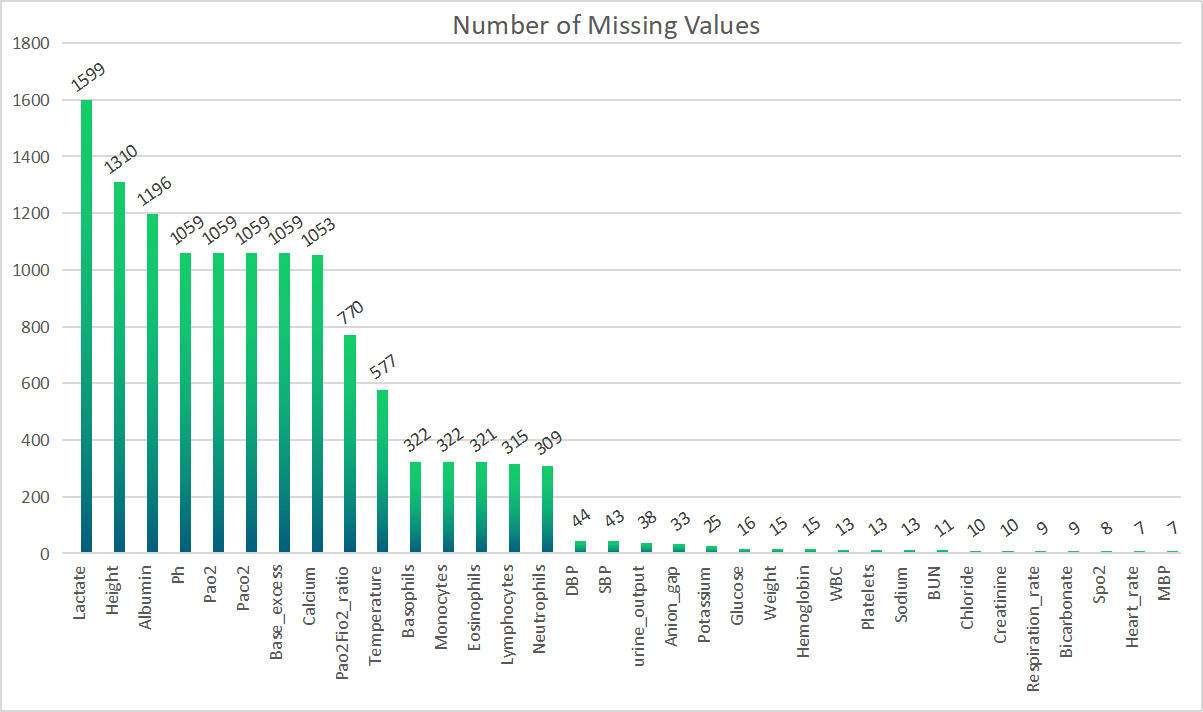


1. Supplementary Figure S1 Number of Missing Values

2. Supplementary Figure S
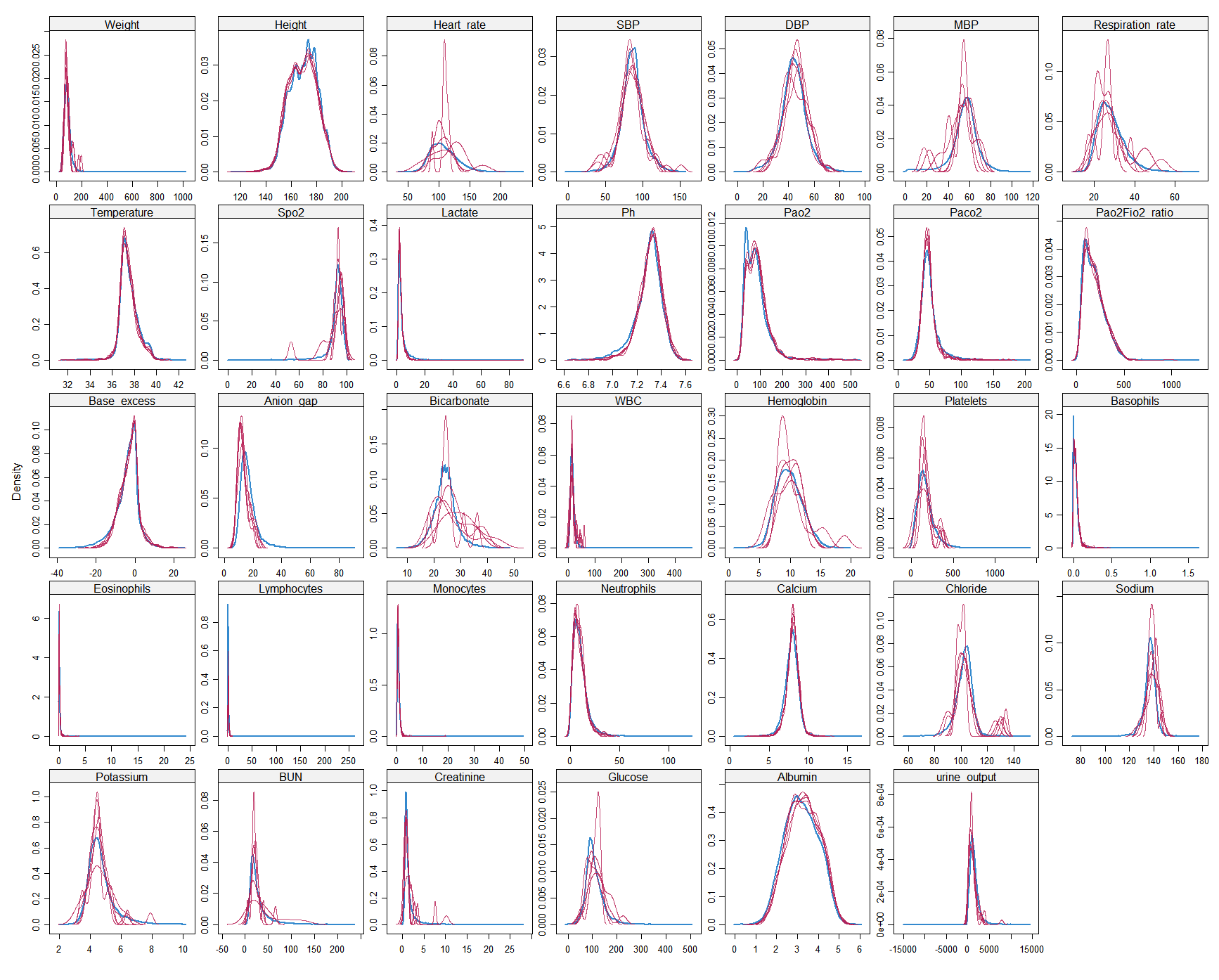
2 Interpolation data distribution map

3. Supplementary Figure S3 The forest plots of u
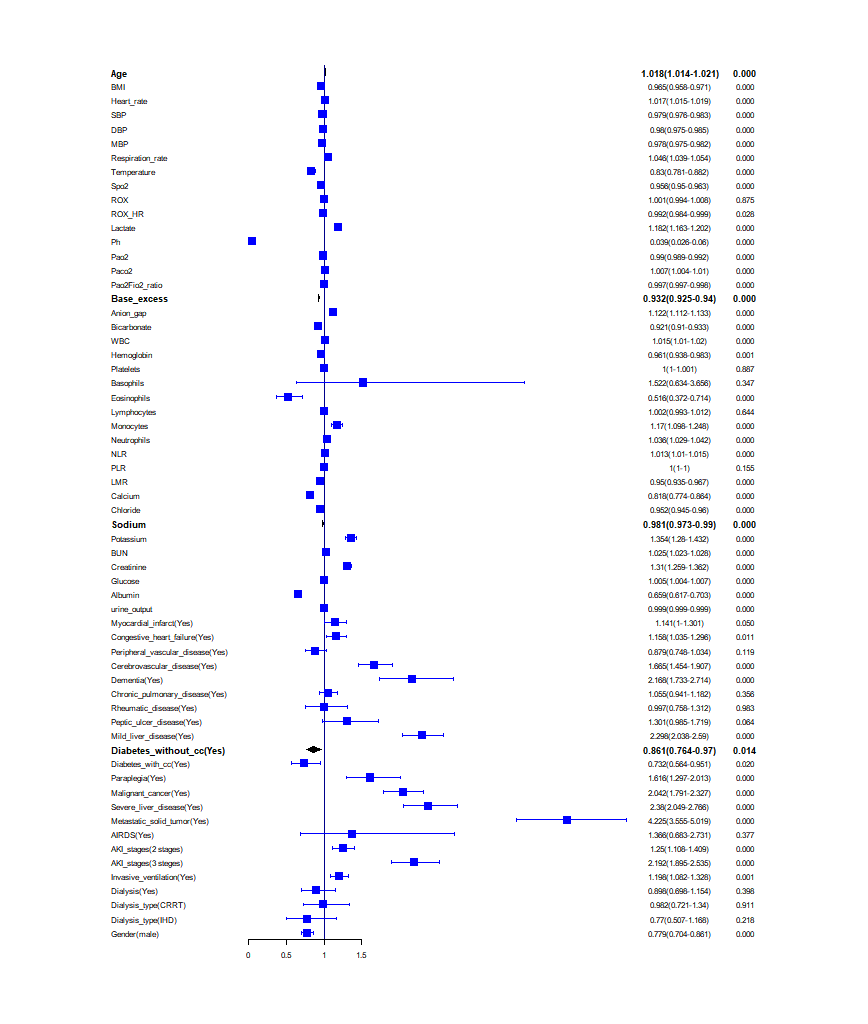
nivariate regression analysis


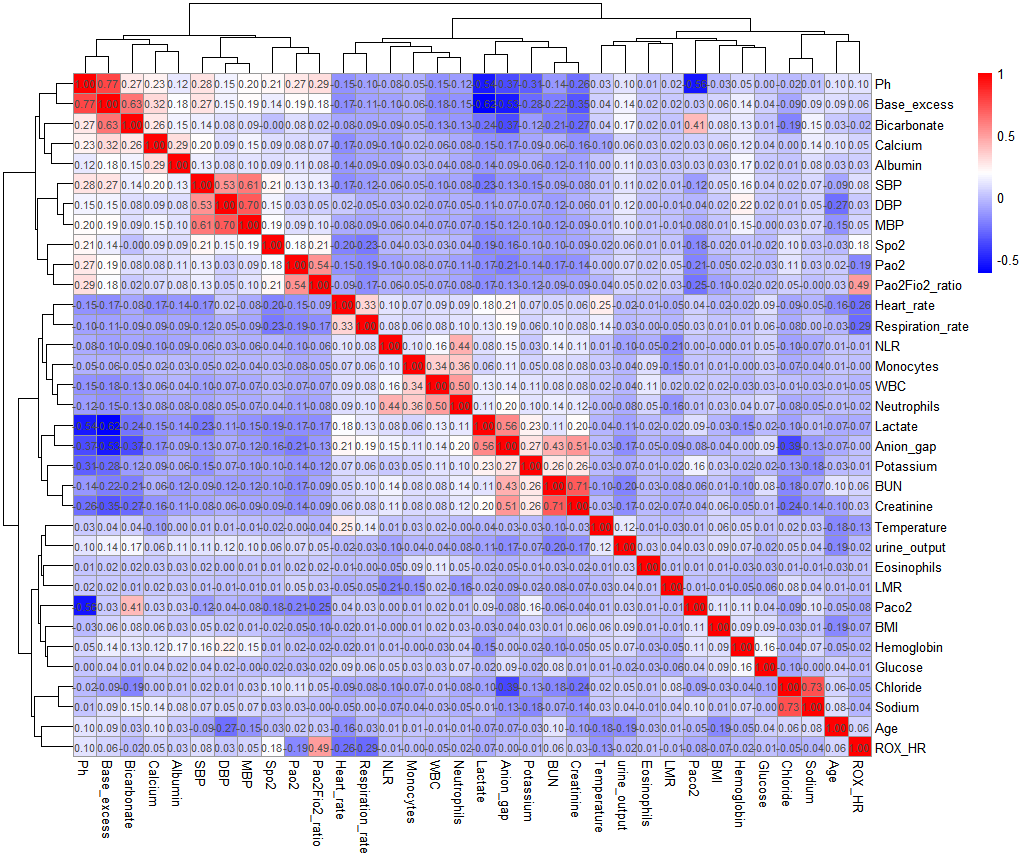


4. Supplementary Figure S4 Correlation heat map between continuous variables


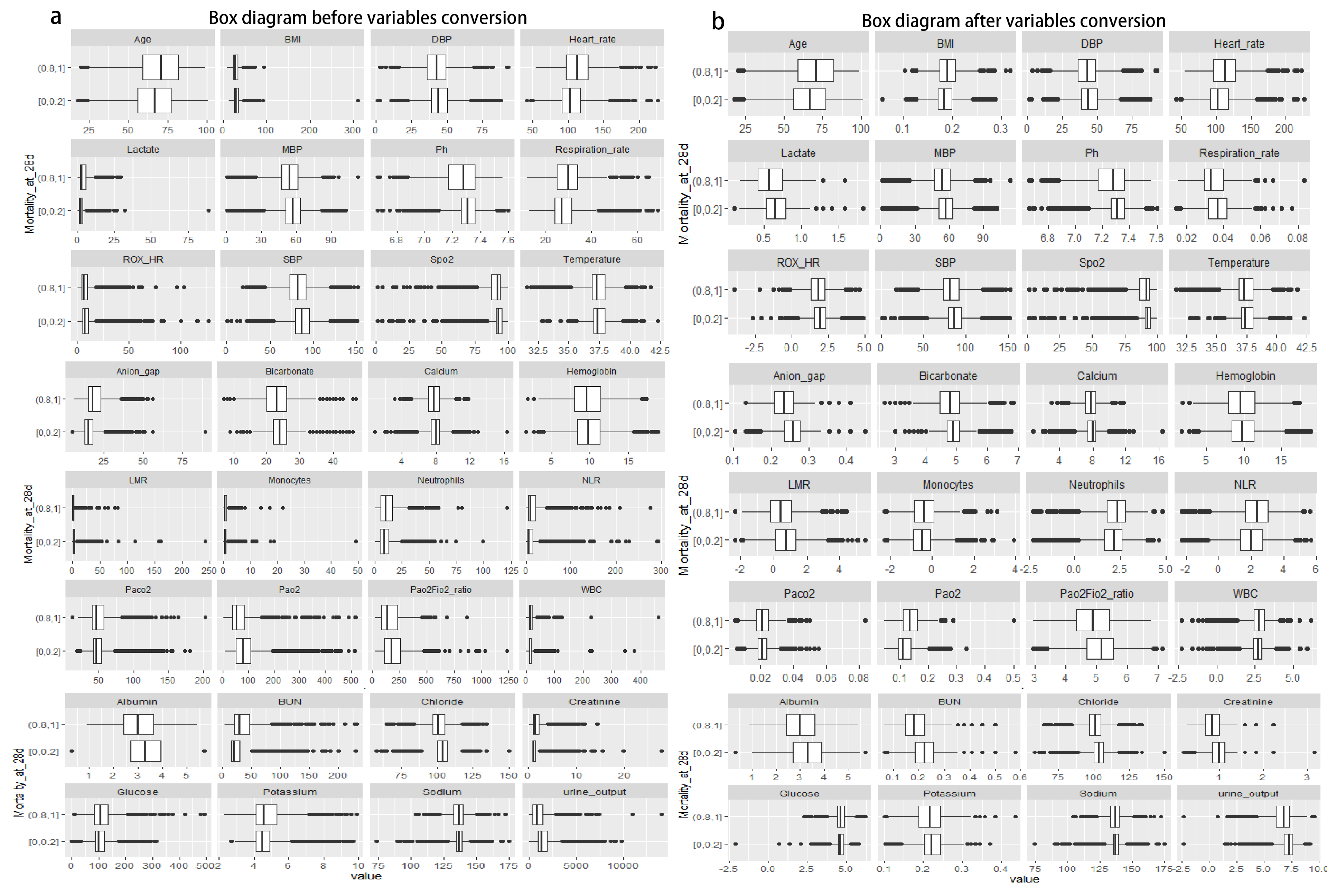


5. Supplementary Figure S5 Variable conversion box diagram
